# High expression of tight junction protein 1 as a predictive biomarker for bladder cancer grade and staging

**DOI:** 10.1038/s41598-022-05631-y

**Published:** 2022-01-27

**Authors:** Yi-Chen Lee, Kuo-Wang Tsai, Jia-Bin Liao, Wei-Ting Kuo, Yu-Chan Chang, Yi-Fang Yang

**Affiliations:** 1 Department of Anatomy, School of Medicine, College of Medicine, Kaohsiung Medical University, Kaohsiung, Taiwan; 2 Department of Medical Research, Kaohsiung Medical University Hospital, Kaohsiung, Taiwan; 3 Department of Research, Taipei Tzu Chi Hospital, Buddhist Tzu Chi Medical Foundation, New Taipei City, Taiwan; 4 Department of Pathology and Laboratory Medicine, Kaohsiung Veterans General Hospital, Kaohsiung, Taiwan; 5 Division of Urology, Department of Surgery, Kaohsiung Veterans General Hospital, Kaohsiung, Taiwan; 6 School of Medicine, National Yang-Ming University, Taipei, Taiwan; 7 Department of Biomedical Imaging and Radiological Sciences, National Yang Ming Chiao Tung University, Taipei, Taiwan; 8 Department of Medical Education and Research, Kaohsiung Veterans General Hospital, No. 386, Dajhong 1st Rd., Zuoying Dist., Kaohsiung, 81362 Taiwan

**Keywords:** Cancer, Functional genomics

## Abstract

Tight junction proteins 1–3 (TJP1–3) are components of tight junctions that can link transmembrane proteins to the actin cytoskeleton, and their incidence directly correlates to metastasis. However, the role of the TJP family in bladder cancer has not been adequately evaluated. In this study, we evaluated the genetic changes, mRNA and protein expressions of the target genes of the TJP family in bladder cancer patients using online database and immunohistochemistry, respectively. We found that *TJP1* was amplified in bladder cancer tissue and that the protein expression levels were significantly associated with age (*p* = 0.03), grade (*p* = 0.007), and stage (*p* = 0.011). We also examined the correlation between *TJP1* and other high-frequency mutation genes using TIMER. *TJP1* mRNA levels were positively correlated with *TTN* and *RYR3* mRNA levels in bladder cancer tissue. Taken together, *TJP1* expression is associated with poor clinical outcomes in patients with bladder cancer and can be a useful predictive biomarker for bladder cancer staging.

## Introduction

Bladder cancer is the common cause of cancer-related deaths worldwide^1,2^. In 2019, there were 30,543 cancer-related deaths in males in Taiwan, of which 711 deaths (2.3%) were from bladder cancer. Bladder cancers are classified into non-muscle-invasive bladder cancer (NMIBC) and muscle-invasive bladder cancer (MIBC)^3^. Urothelial carcinoma is the most common type of bladder cancer. Approximately 50–70% of patients with NMIBC show recurrence after treatment and of those, 10–15% of patients develop MIBC, which are invasive malignant tumors that have a 50–60% survival rate (5-year)^4,5^. When the MIBC progresses into metastatic bladder cancer, the 5-year survival rate of patients is significantly reduced^4^. Locally advanced or metastatic bladder cancer has a high mortality rate, having only a 5% 5-year survival rate in patients with bladder cancer in the last stage^6,7^. Furthermore, tumor grade as an important prognostic indicator in NMIBC and increasing tumor grade was associated with higher disease progression and recurrence rates^1^. However, the operative mechanisms of how cancer cells modulate the malignant phenotype underlying the disease remain unclear and must be further investigated to effectively improve the poor prognosis of bladder cancer.

Tight junctions (TJs), adherens junctions (AJs), and desmosomes are protective barriers for epithelial and endothelial cells that serve as sentries in the living system^8–10^. TJ barrier functions include the regulation of intercellular communication and paracellular transport^11^. TJ proteins include claudins, occludins, and framework forming proteins: cinguline, PALS1 (protein associated with Lin Seven 1), MUPP1 (multi-PZD domain protein 1), and ZO-1 (TJP1), ZO-2 (TJP2), and ZO-3 (TJP3) (zona occludens)^12^. ZO proteins play an important role in the formation of tight junctions that directly interact with the PDZ domain (ZO-1 or ZO-2) and the C-terminus of claudins^13^. Moreover, these proteins modulate several signaling pathways in cancer cells. Previous studies show that the downregulation of ZO-1 leads to increased motility in pancreatic cancer^14^. However, the upregulation of ZO-1 contributes to the invasion and adhesion of melanoma cells^15^. Accumulating evidence suggests that ZO proteins play a central role in cancer progression. However, the role of ZO proteins in bladder cancer has not been elucidated.

In this study, we investigated whether the ZO family (TJP ZO1, ZO-2, and ZO-3, encoded by the *TJP1*, *TJP2,* and *TJP3* genes, respectively) is associated with the malignant phenotype in bladder cancer. We examined the DNA copy number and mRNA expression of the ZO family in multiple cancer cell lines and cancer patients using online datasets. Based on the analysis of the bioinformation, we identified the target genes and then provided the clinicopathological data for verification relationships in patients with bladder cancer.

## Results

### In silico mRNA and DNA profiles of TJP family members in multiple cancer cell lines

**Figure 1:**
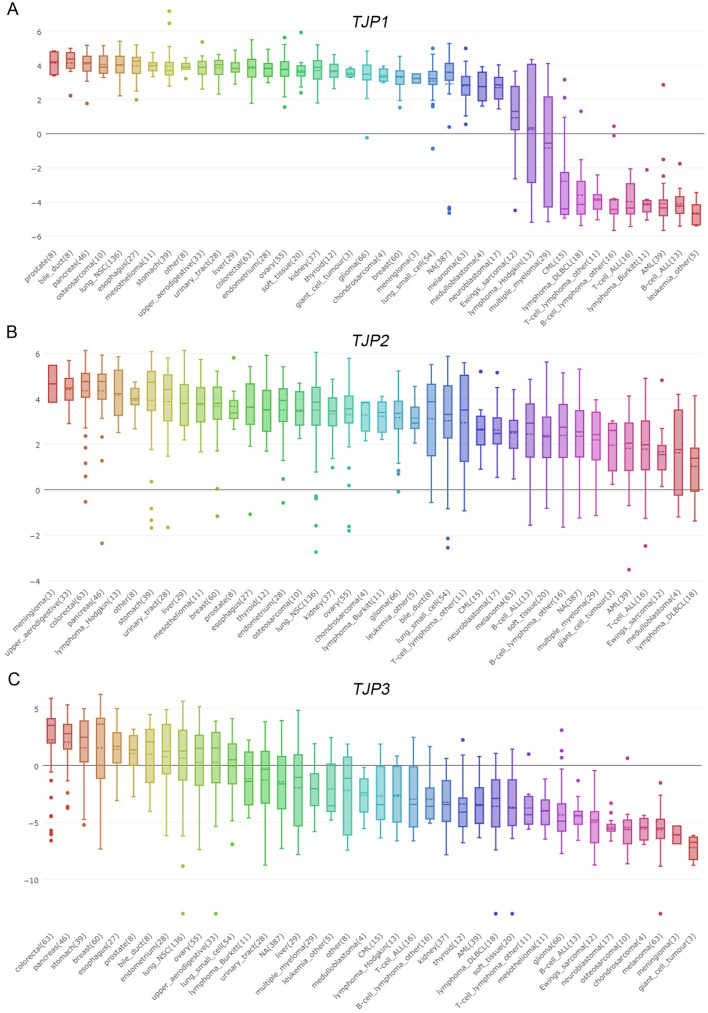
TJP family mRNA expression in multiple cancer cell lines. Relative *TJP1* (**A**), *TJP2* (**B**) and *TJP3* (**C**) mRNA expression in multiple cancer cell lines (CCLE dataset). The number of parentheses that how many cell have in the same cancer type.

**Figure 2:**
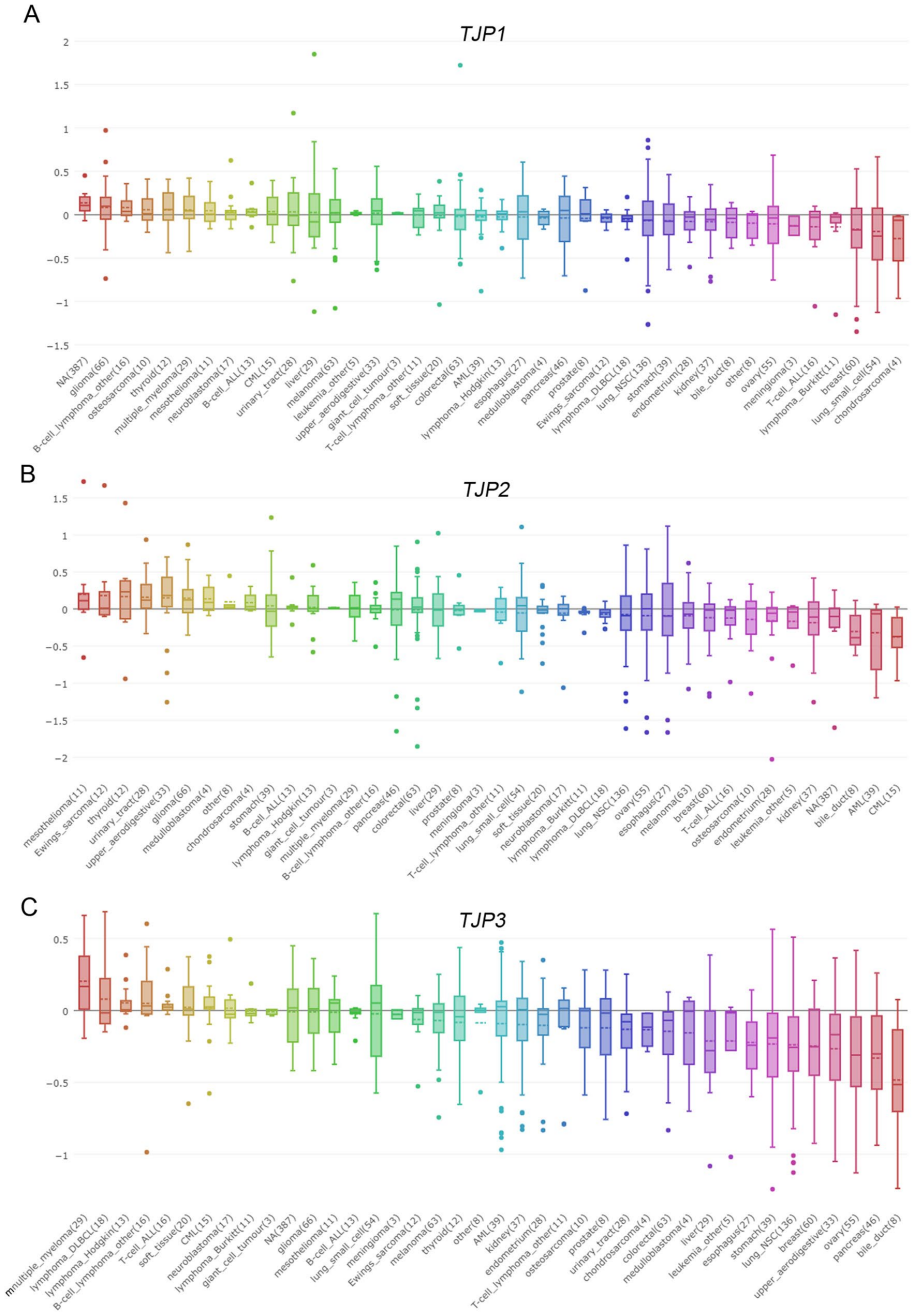
TJP1 DNA copy number in multiple cancer cell lines. Relative *TJP1* (**A**), *TJP2* (**B**) and *TJP3* (**C**) DNA copy number in multiple cancer cell lines (CCLE dataset). The number of parentheses that how many cell have in the same cancer type.

By evaluating the mRNA expression of *TJP1*, *TJP2*, and *TJP3* in 40 different cancer cell lines, we found that *TJP1*, *TJP2,* and *TJP3* were upregulated in 29, 40, and 12 different cancer cell types, respectively (Fig. 1). Next, by examining the DNA copy numbers of the TJP family in multiple cancer cell lines, we observed that the DNA copy numbers of *TJP1*, *TJP2,* and *TJP3* were upregulated in 10, 9, and 7 different cancer cell lines, respectively (Fig. 2). However, the DNA copy numbers and mRNA expression levels were not consistent.

### In silico genetic alterations of TJP family members in bladder cancer patients

**Figure 3:**
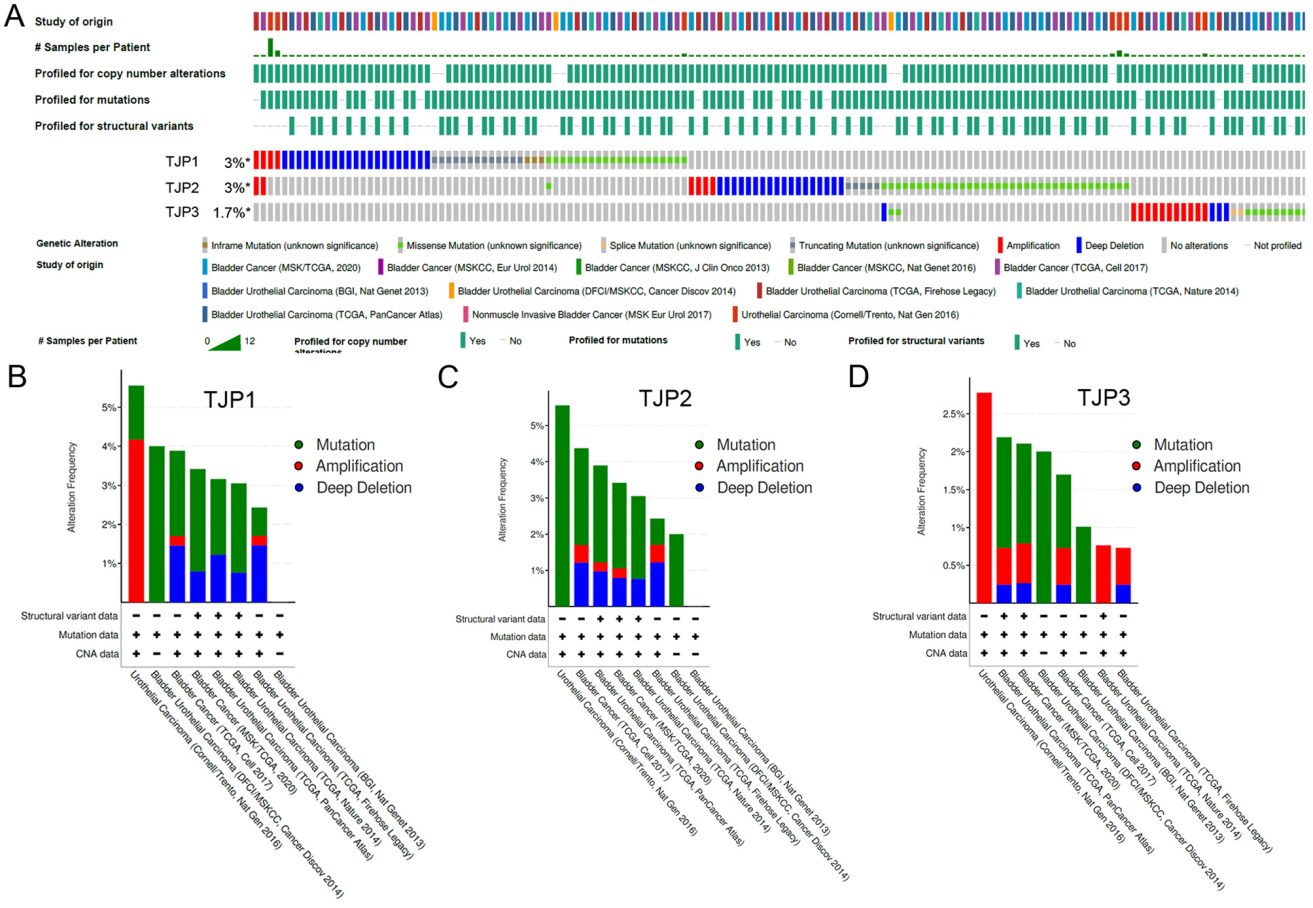
TJP1 amplification in bladder cancer. (**A**) Oncoprint showing *TJP1*, *TJP2* and *TJP3* genetic alterations in bladder cancer patients, respectively. Colors indicated type of genetic alteration (missense, inframe, truncated, amplification, deletion, fusion) and different cohort in the below the oncoprint. (**B**–**D**) Cancer type summary of *TJP1* (**B**), *TJP2* (**C**) and *TJP3* (**D**) by different bladder cancer cohorts. *CAN* copy number alterations.

The genetic alterations of the TJP family in bladder cancer, examined using cBioPortal, showed that the genetically altered ratios of *TJP1*, *TJP2,* and *TJP3* were 3%, 3%, and 1.7%, respectively (Fig. 3A). *TJP1* and *TJP3* had a 2.5–4% amplification ratio and *TJP2* has a 5% mutation ratio in bladder cancer patients (Fig. 3B–D). While analyzing the frequency of the co-occurrence of genetically altered TJP1–3 in the same specimen, it was found that genetic alterations of the other TJP family members were not significant in bladder cancer patients (Supplementary Table 1). *TJP1* genetic amplification was correlated with mRNA expression in patients with bladder cancer (Supplementary Fig. 1A). In addition, we also compared the TJP1 expression between the protein level and the RNA level from the CCLE dataset (https://depmap.org/portal/). Our results found a strong positive correlation in the bladder cancer cell panel (Spearman nonparametric correlation test; correlation coefficient = 0.582; *p* = 0.05, n = 11) (Supplementary Fig. 1B). Moreover, we further evaluated expression of TJP1 protein in bladder cancer cell lines. The results showed TJP1 protein was upregulated in bladder cancer cells (Supplementary Fig. 1C). A previous study showed that TJP1 expression is correlated with cell motility in bladder cancer cells^16^. Therefore, in this study, we focused on TJP1 for further investigation of bladder cancer.

### TJP1 protein up-regulation is associated with poor clinical outcomes in bladder cancer

**Figure 4:**
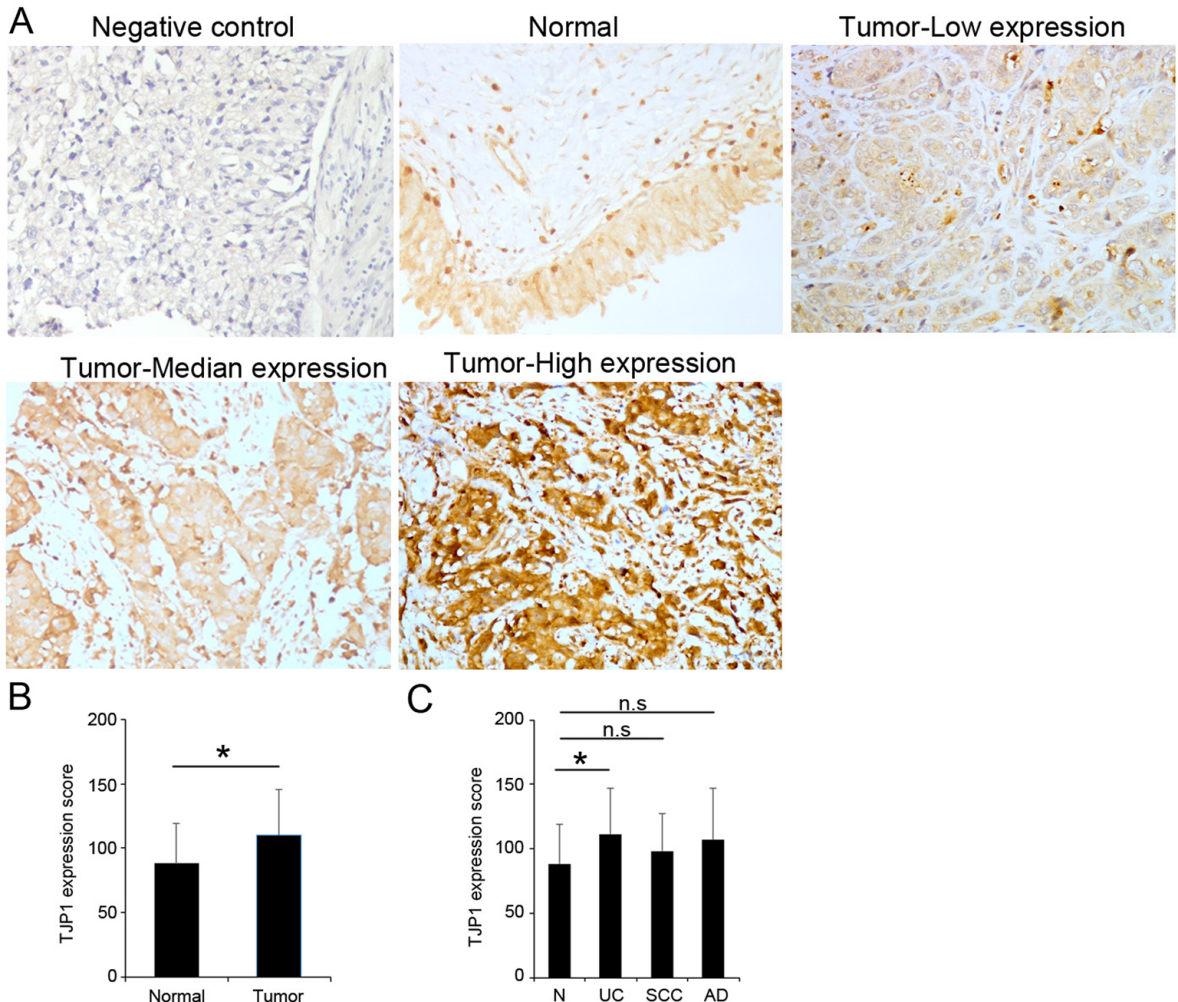
TJP1 upregulated in bladder cancer. (**A**) Representative IHC staining images of TJP1 in bladder cancer tissues. (**B**) Relative TJP1 protein levels in adjacent non-tumor tissue (n = 16) and bladder cancer tissues (n = 192). (**C**) Relative TJP1 protein levels in urothelial carcinoma (UC, n = 160), squamous cell (SCC, n = 16) and adenocarcinoma (AD, n = 16) in bladder cancer tissues. Data are presented as the mean ± SD, *p < 0.05. *ns* not significant.

As shown in Fig. 4A, TJP1 expression in cancer tissues was classified into two groups (low and high) base on cutoff point, which was set at the median. It was found that high TJP1 expression levels in bladder cancer tissues were significantly associated with age (*p* = 0.03), grade (*p* = 0.007), and stage (*p* = 0.011) (Table 1). IHC staining results show that TJP1 was significantly upregulated in bladder cancer specimens compared to normal bladder tissues (Fig. 4B). The results also showed that TJP1 was significantly upregulated in the urothelial carcinoma group in bladder cancer specimens compared to normal bladder tissues (Fig. 4C).

**Table 1:** Correlation of TJP-1 expression with clinicopathological characteristics in bladder cancer.

Variables	Item	Patient no. (%)	TJP-1				*p* value
			Low (≤ 108.33)		High (> 108.33)		
			No.	%	No.	%	
		192 (100.0)	96	50.0	96	50.0	
Age (y)	≤ 60	89 (46.4)	52	54.2	37	38.5	0.030
	> 60	103 (53.6)	44	45.8	59	61.5	
Sex	Female	39 (20.3)	16	16.7	23	24.0	0.209
	Male	153 (79.7)	80	83.3	73	76.0	
Grade	I	64 (33.9)	40	42.1	24	25.5	0.007
	II	81 (42.9)	41	43.2	40	42.6	
	III	44 (23.3)	14	14.7	30	31.9	
Stage	I/II	154 (80.2)	84	87.5	70	72.9	0.011
	III/IV	38 (19.8)	12	12.5	26	27.1	
Histology	Urothelial carcinoma	160 (83.3)	79	82.3	81	84.4	0.194
	Squamous cell carcinoma	16 (8.3)	11	11.5	5	5.2	
	Adenocarcinoma	16 (8.3)	6	6.2	10	10.4	

### *TJP1* expression positive correlates with *TTN* in bladder cancer patients

**Figure 5:**
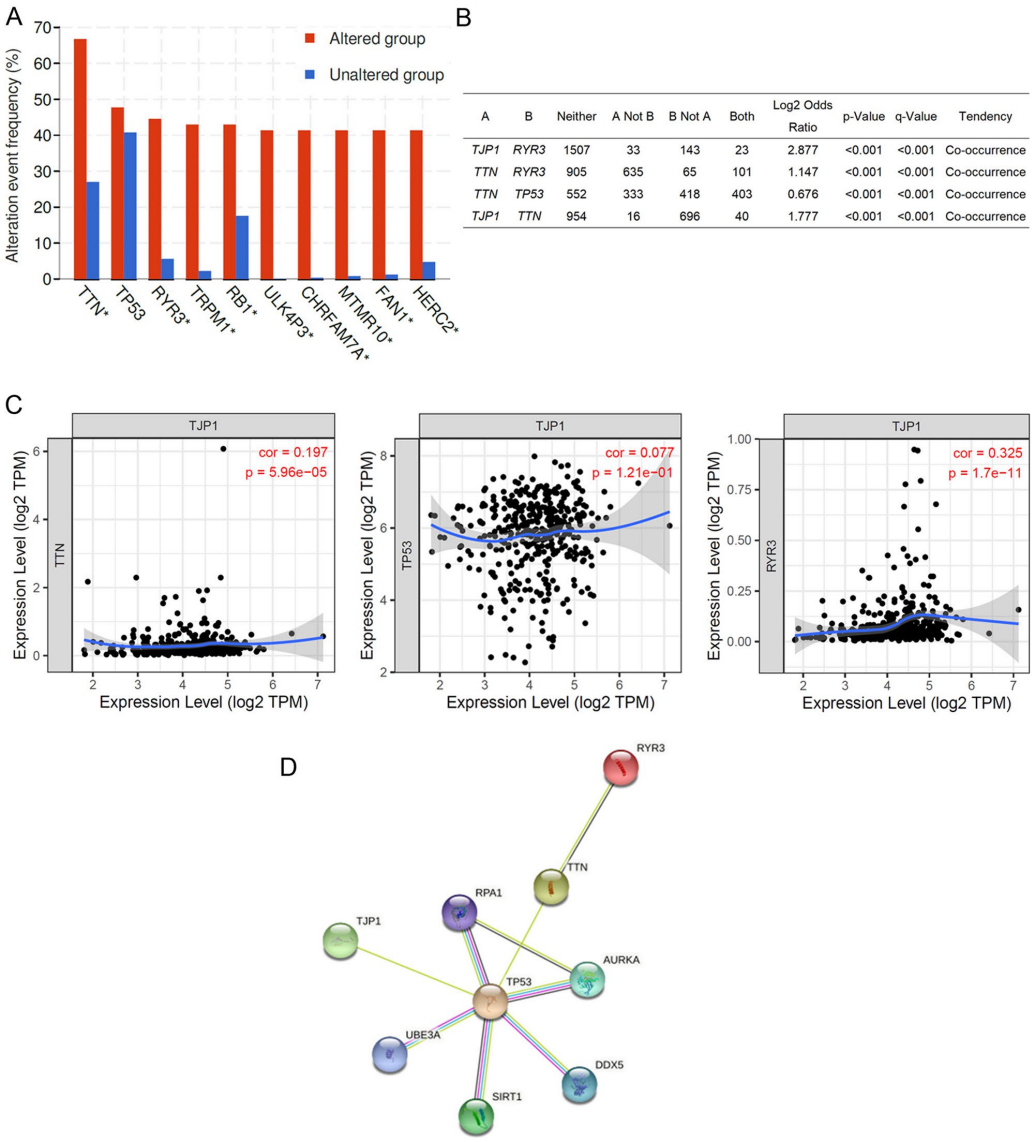
Correlation analysis of *TJP1* and oncogenes in bladder cancer patients. (**A**) Frequency of genomic alteration in tumors from bladder cancer patients. *p < 0.05. (**B**) Major co-occurrences of genomic alterations of *TJP1* and *TTN, TP53* and *RYR3* in bladder cancer patients. (**C**) Analysis of the correlation between *TJP1* and *TTN*/*TP53*/*RYR3* mRNA expression using TIMER. (**D**) STRING protein–protein interaction networks for TJP1, TP53, TTN and RYR3.

It was observed that *TTN, RYR3, TRPM1, RB1, ULK4P3, CHRFAM7A, FAN1*, and *HERC2* were significantly altered in patients with bladder cancer (Fig. 5A). Furthermore, Fig. 5B shows that *TJP1* genetically altered co-occurrence in a series of core genes, including *TTN, TP53*, and *RYR3.* We further examined the correlation between the mRNA expression of *TJP1* and *TTN, TP53*, and *RYR3* by TIMER^17^. The results showed that *TJP1* mRNA levels were positively correlated with *TTN* and *RYR3* mRNA levels in bladder cancer tissues (Fig. 5C). Moreover, we examined the protein–protein interactions network by using STRING dataset^18^. The results showed TJP1 interaction with TTN and RYR3 via TP53 (Fig. 5D and Supplementary Table 2).

### TJP family expression correlated with chemotherapy response in bladder cancer cells

It has been shown that genetic determinants for chemotherapy and radiotherapy response in bladder cancer^19^. To examine the correlation between TJP family mRNA expression and chemotherapy drugs in bladder cancer cells. We found *TJP2* expression was positively correlated with IC50 of cisplatin, and *TJP3* expression was positively correlated with IC50 of mitomycin C in bladder cancer cell lines (Table 2 and Supplement Figs. 2–4).

**Table 2:** Correlation of TJP family expression with chemotherapy drugs in bladder cancer cell lines.

Drugs	*TJP1*		*TJP2*		*TJP3*	
	Correlation	*p*-value	Correlation	*p*-value	Correlation	*p*-value
Cisplatin	0.427	0.095	0.591	0.028*	0.345	0.149
Doxorubicin	0.082	0.405	0.264	0.217	0.145	0.335
Epirubicin	− 0.082	0.405	− 0.127	0.355	0.055	0.437
Gemcitabine	− 0.200	0.278	0.291	0.193	0.336	0.156
Mitomycin C	0.373	0.129	0.291	0.193	0.555	0.038*

*p values <0.05 were considered statistically significant.

## Discussion

TJP1, also known as zona occludens 1 (ZO-1), is a tight junction protein that can regulate actin cytoskeleton remodeling^20^. Altered expression of *TJP1* is found in many cancers and is responsible for modulating cancer migration and invasion^15,20–22^. In this study, the TJP family was evaluated in multiple cancer cell lines and it is a predictive biomarker for bladder cancer staging.

Our data showed that *TJP1* is upregulated in multiple cancer cell lines (Fig. 1A). TJP expression regulates several signaling and transcriptional pathways in cancer^23^ and is involved in the epithelial-mesenchymal transition (EMT) associated with tumor invasion^24^. Downregulation of *TJP1* expression has been observed in gastrointestinal adenocarcinoma, breast cancer, and colorectal carcinoma^25–27^. *TJP1* expression is regulated by E-cadherin in breast carcinoma^26^. During EMT, downregulation of E-cadherin is accompanied by the upregulation of N-cadherin expression, which promotes cell motility and survival advantage in the early stage tumor^28,29^. In contrast, Smalley et al. showed that TJP1 is upregulated and co-localized with N-cadherin and contributes to adhesion and invasion abilities in the melanoma cell^15^. Specifically, high *TJP1* mRNA expression has been reported in patients with bladder cancer. Knockdown of TJP1 inhibits cell proliferation, migration, and invasion in bladder cancer cell lines, while, *TJP1* mRNA expression is associated with lymph node metastasis in bladder cancer patients^16^. Additionally, deletions and mutations of *TJP1* promote cancer cell proliferation^25^. In our data showed expression of TJP1 protein was associated with grade and stage in patients with bladder cancer (Table 1).

Previous studies have shown that tumor mutational burden is a biomarker for predicting responsiveness to immune checkpoint blockade immunotherapy in several cancer types^30,31^. In our study, we found that *TNT*, *TP53,* and *RYR3* mutations co-occurred with altered *TJP1* in bladder cancer patients. In addition, *TJP1* mRNA expression levels were positively correlated with *TNT and RYR3* mRNA expression levels in patients with bladder cancer. We could not exclude the possibility that *TJP1* amplification or expression is correlated with the response rate to immune checkpoint blockade; however, this is the first study to evaluate the *TJP1* genetic alterations in bladder cancer patients. Accumulating evidence has shown genetic altered and expression correlated with chemotherapy response in bladder cancer^19^. We also found *TJP2* and *TJP3* mRNA expression positively correlated with chemoresistance in bladder cancer cell lines (Table 2). However, *TJP1* mRNA expression was not significantly correlated with chemotherapy in bladder cancer cell lines. Further mechanisms are needed to investigate the underlying TJP1 expression and subsequently increased chemosensitivity in bladder cancer.

In conclusion, this study evaluated genetic variations in the *TJP1* family by the amplification of TJP family members in bladder cancer patients. TJP1 protein expression correlated to tumor grade and stage, indicating that TJP1 can be used as an independent biomarker for bladder cancer staging.

## Materials and methods

### In silico genetic and mRNA profiles of the TJP1 family in multiple cancer cell lines and cancer patients

The mRNA expression levels and DNA copy numbers of *TJP1*, *TJP2*, and *TJP3* in 40 different cancer cell lines were evaluated using the Cell Line Encyclopedia (CCLE) dataset. We stratified cancer cell types into upregulated (median > 0) and downregulated (median < 0). Genetic variations in the TJP1 family and genetic altered ranking in bladder cancer patients were evaluated using the online dataset (cBioPortal, v.3.6.20).

### Patients and specimen collection

Bladder tumor tissue was collected from 192 patients with bladder cancer (sample from 160 patients with urothelial carcinoma, 16 patients with squamous cell and 16 patients with adenocarcinoma). The bladder cancer tissue array (#BL2081a) was purchased from Biomax (Rockville, MD, USA) and was used for immunohistochemical (IHC) staining to evaluate the expression of TJP1 protein. The pathologic grade was classified based on the World Health Organization (WHO) histological criteria^32^.

### Immunohistochemical staining

IHC staining was performed according to the manufacturer's instructions. The primary antibody of TJP1 (#HPA001636, 1:50) was purchased from Sigma-Aldrich (St. Louis, MO, USA). TJP1 expression was evaluated using the H-score, which is calculated as the percentage of positively stained cells multiplied by the staining intensity. In this study, two physicians (Y.-C L and J.-B L) were used to objectively evaluate the scores.

### Correlation of *TJP1* with other high frequency mutation genes

The genetic alteration, co-occurrence, and mRNA expression levels of high-frequency mutation genes (*TTN, RYR3, TRPM1, RB1, ULK4P3, CHRFAM7A, FAN1*, and *HERC2)* correlating to TJP1 expression were evaluated using TIMER.

### Correlation of *TJP* family with chemotherapy drugs

*TJP* family mRNA levels downloaded from depmap portal (https://depmap.org/portal/download/?release=CCLE+2019&release=Fusion&release=DNA+Copy+Number). The IC50 of cisplatin, doxorubicin, epirubicin, gemcitabine, mitomycin C was downloaded from Genomics of Drug Sensitivity in Cancer database (https://www.cancerrxgene.org/). The bladder cancer cell lines include 5637, 639V, 647V, HT1197, HT1376, KU1919, RT-122, T24, TDDSUP, UMUC3, and VMCUB1.

### Statistical analyses

All statistical analyses were performed using SPSS (version 19.0; IBM, Armonk, NY, USA). The Chi-squared test was used to determine the correlation between TJP1 expression and the tumor stage, grade, size, and the patient’s age at diagnosis. The Student’s *t* test was used to identify significant differences between the treatment groups. Statistical significance was set at *p* < 0.05.

## Supplementary Information

Supplementary Information.
