# Supplementary material for: High expression of tight junction protein 1 as a predictive biomarker for bladder cancer grade and staging
Source: Sci Rep. 2022 Jan 27;12:1496. doi: 10.1038/s41598-022-05631-y (PMC8795112; doi:10.1038/s41598-022-05631-y)
Supplement: Supplementary file 1 — Supplementary Information. [file 41598_2022_5631_MOESM1_ESM.docx]

High expression of tight junction protein 1 as a predictive biomarker for bladder cancer grade and staging

Yi-Chen Lee^1,2^, Kuo-Wang Tsai^3^, Jia-Bin Liao^4^, Wei-Ting Kuo^5,6^, Yu-Chan Chang^7^ and Yi-Fang Yang^8,*^

https://orcid.org/0000-0002-4889-095X (Yi-Chen Lee)

https://orcid.org/0000-0002-9028-9834 (Kuo-Wang Tsai)

https://orcid.org/0000-0003-0474-9935 (Yu-Chan Chang)

https://orcid.org/0000-0001-7425-3156 (Yi-Fang Yang)

^1^Department of Anatomy, School of Medicine, College of Medicine, Kaohsiung Medical University, Kaohsiung, Taiwan

^2^Department of Medical Research, Kaohsiung Medical University Hospital, Kaohsiung, Taiwan

^3^Department of Research, Taipei Tzu Chi Hospital, Buddhist Tzu Chi Medical Foundation, New Taipei City, Taiwan

^4^Department of Pathology and Laboratory Medicine, Kaohsiung Veterans General Hospital, Kaohsiung, Taiwan

^5^Division of Urology, Department of Surgery, Kaohsiung Veterans General Hospital, Kaohsiung, Taiwan

^6^School of Medicine, National Yang-Ming University, Taipei, Taiwan

^7^ Department of Biomedical Imaging and Radiological Sciences, National Yang- Ming Chiao Tung University, Taipei, Taiwan

^8^Department of Medical Education and Research, Kaohsiung Veterans General Hospital, Kaohsiung, Taiwan

Correspondence to: Yi-Fang Yang, PhD

Department of Medical Education and Research, Kaohsiung Veterans General

Hospital, Kaohsiung, Taiwan, No. 386, Dajhong 1st Rd., Zuoying Dist., Kaohsiung City 81362, Taiwan. Phone: 886-7-342-2121 # 71592; Fax: 886-7-342-2288; E-mail: yvonne845040@gmail.com

**Running Title**: TJP1 associated bladder cancer staging

**Keywords**:

TJP1, bladder cancer, stage, grade

**Conflict of interest:** The authors declare that they have no conflicts of interest.

Inventory of all Supplemental Information

Supplemental materials and methods

Supplemental Data

Figure S1 Related to Figure 3

Figure S2

Figure S3

Figure S4

Supplementary Tables

Supplementary Table 1 Related to Figure 3

Supplementary Table 2 Related to Figure 5

**Supplemental materials and methods**

**Cell lines and cell culture conditions**

The urothelial carcinoma cell lines 5637 and T24 were maintained in DMEM with 10% FBS and 1% Penicillin-Streptomycin-Glutamine (PSG). BFTC-909 was maintained in DMEM with 10% FBS, 1%sodium pyruvate and 1% PSG. UM-UC-14 was maintained in DMEM with 10% FBS, 1%NEAA, 1%sodium pyruvate and 1% PSG. BFTC-905 was maintained in DMEM with 15% FBS and 1% PSG. RT-4 was maintained in McCoy’s 5A with 10% FBS and 1% PSG.

**Western blot analysis**

Protein (15μg) was loaded into 10% SDS-polyacrylamide gels and the proteins were transferred to a nitrocellulose membrane after electrophoresis. Immunoblotting was performed using the TJP1 (#HPA001636, 1:1000, Sigma-Aldrich, MO, USA) or GAPDH (#GTX100118, 1:5000, GeneTex, USA).

**Representative image of western blot analysis.**


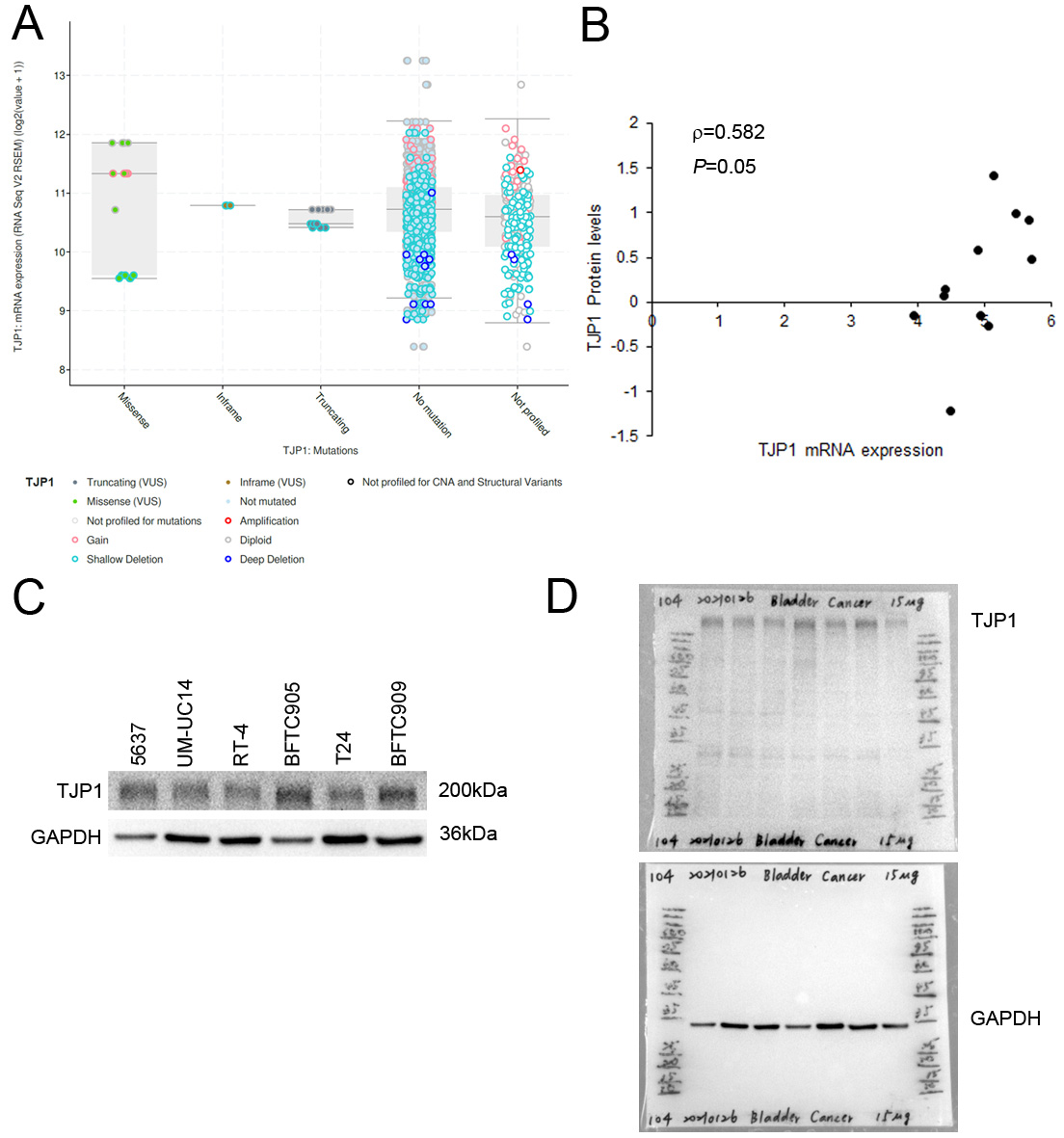


**Supplement Figure1.**


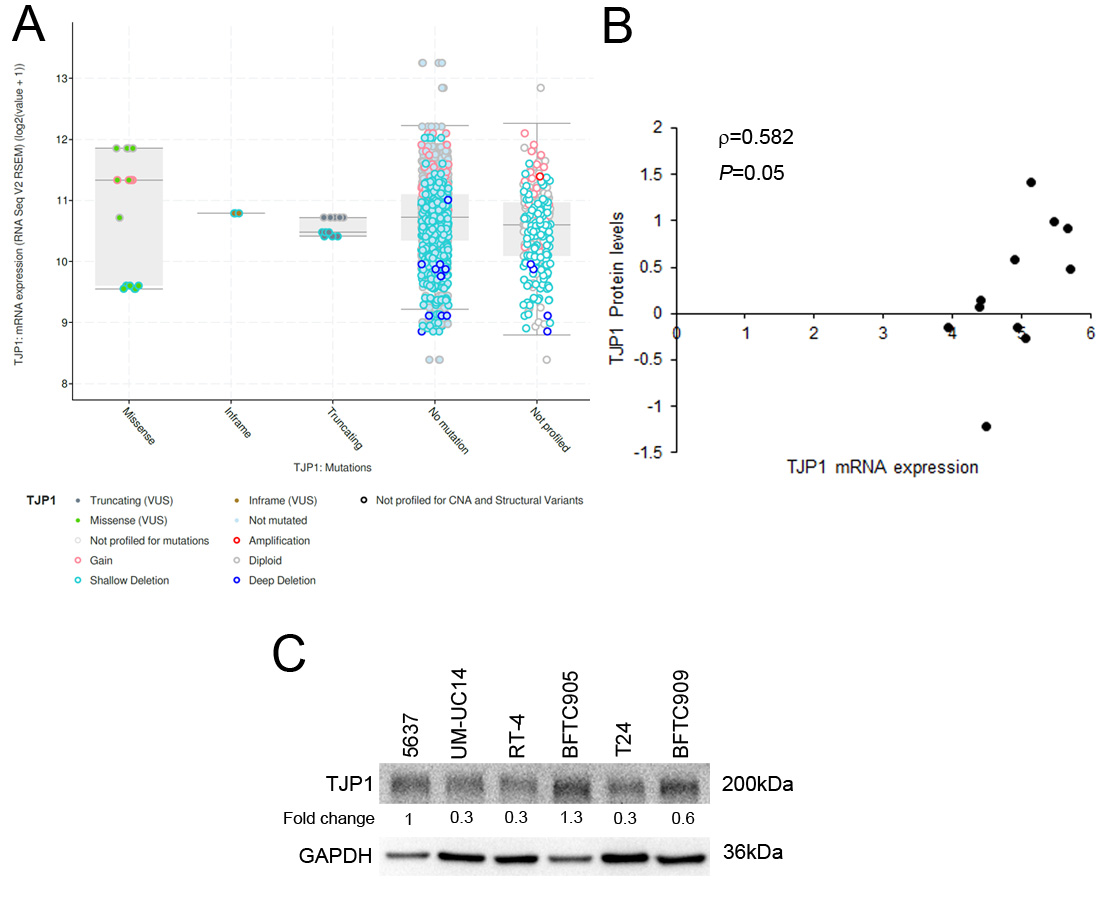


**Supplement Figure1.** (A) Relative correlation of *TJP1* with DNA copy number and mRNA expression in bladder cancer patients as reported by cBioPortal. (B) Analysis of the correlation between protein and mRNA expression of TJP1 in bladder cancer cell lines using CCLE database (n=11). (C) Western blot analysis of TJP1 in bladder cancer cell lines.

**Supplement Figure2.**


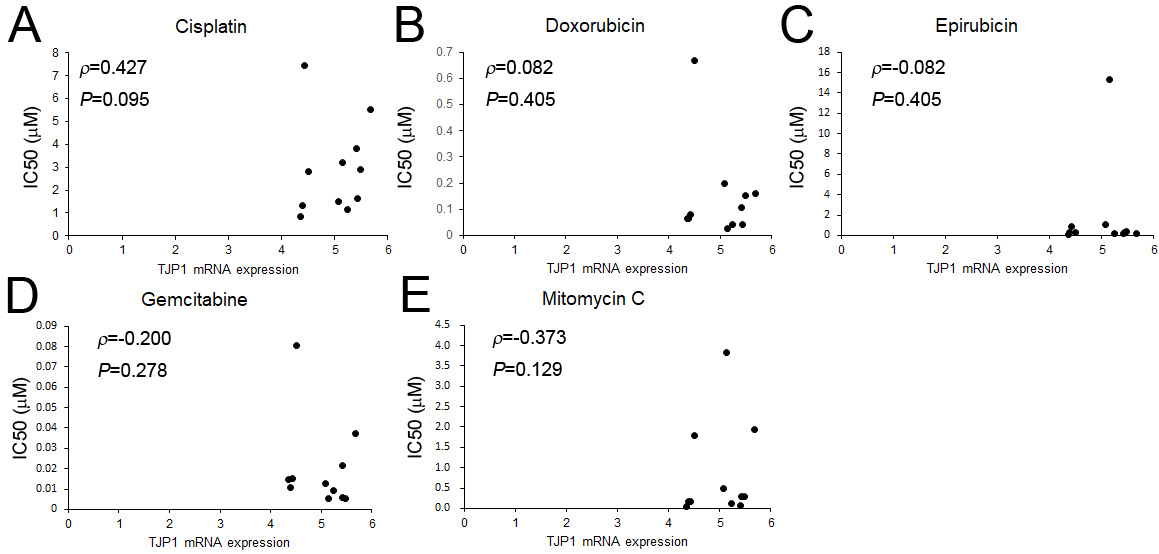


**Supplement Figure2.** The correlation between *TJP1* mRNA expression and cisplatin (A), doxorubicin (B), epirubicin (C), gemcitabine (D), mitomycin C (E) in bladder cancer cell lines.

**Supplement Figure3.**


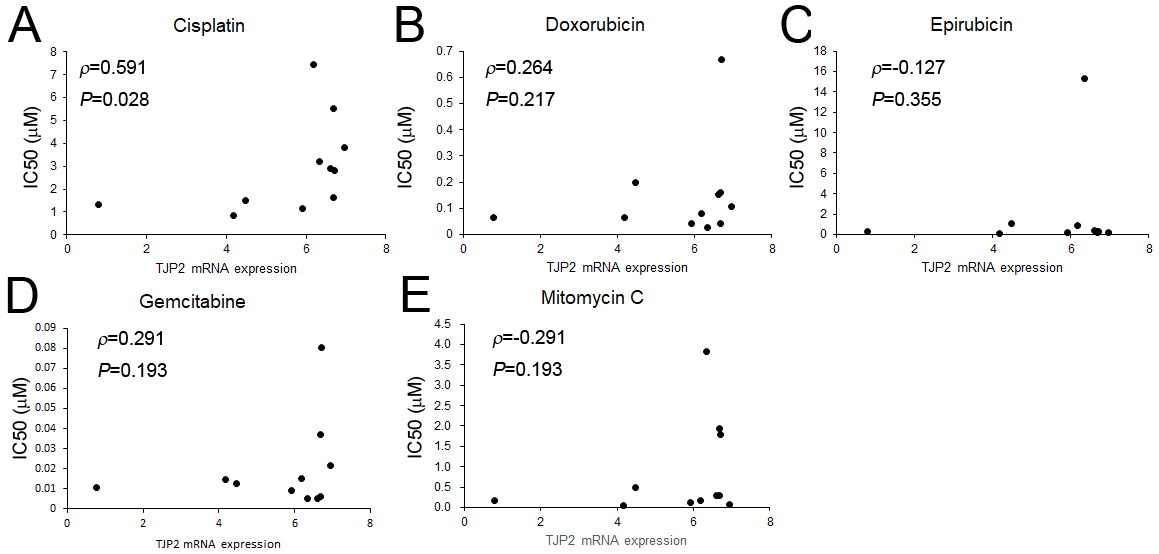


**Supplement Figure3.** The correlation between *TJP2* mRNA expression and cisplatin (A), doxorubicin (B), epirubicin (C), gemcitabine (D), mitomycin C (E) in bladder cancer cell lines.

**Supplement Figure4.**


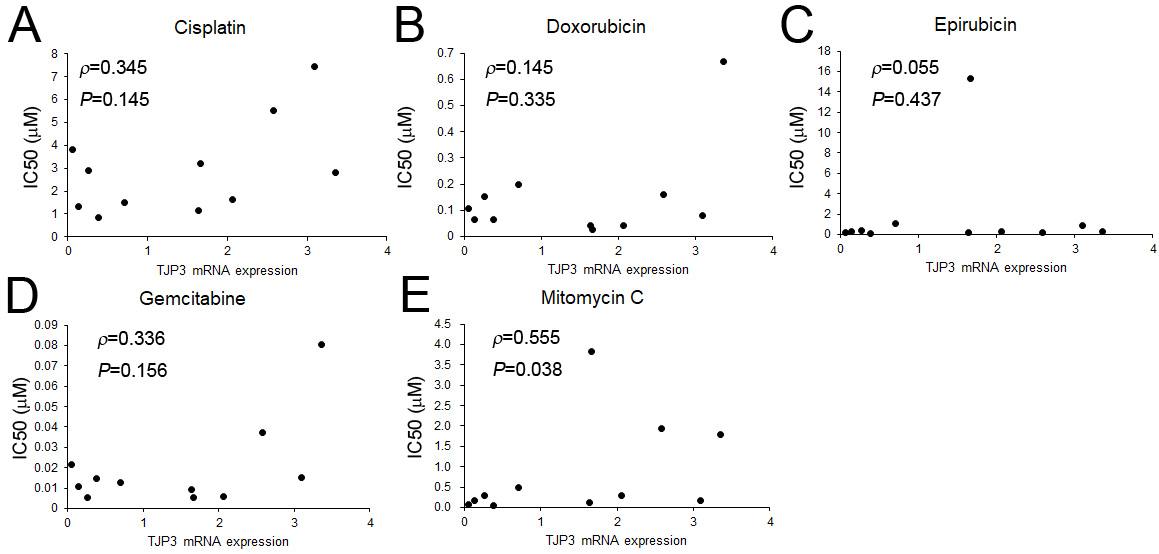


**Supplement Figure4.** The correlation between *TJP3* mRNA expression and cisplatin (A), doxorubicin (B), epirubicin (C), gemcitabine (D), mitomycin C (E) in bladder cancer cell lines.

Supplementary Table 1. TJP family mutual exclusivity

| **A** | **B** | **Neither** | **A Not B** | **B Not A** | **Both** | **Log2 Odds Ratio** | **p-Value** | **q-Value** | **Tendency** |
| --- | --- | --- | --- | --- | --- | --- | --- | --- | --- |
| *TJP2* | *TJP3* | 1622 | 55 | 27 | 2 | 1.127 | 0.252 | 0.565 | Co-occurrence |
| *TJP1* | *TJP3* | 1621 | 56 | 29 | 0 | <-3 | 0.377 | 0.565 | Mutual exclusivity |
| *TJP1* | *TJP2* | 1595 | 54 | 55 | 2 | 0.103 | 0.566 | 0.566 | Co-occurrence |

Supplementary Table 2. STRING interactions short

| #node1 | node2 | node1_string_id | node2_string_id | coexpression | experimentally_determined_interaction | database_annotated | automated_textmining | combined_score |
| --- | --- | --- | --- | --- | --- | --- | --- | --- |
| AURKA | RPA1 | 9606.ENSP00000216911 | 9606.ENSP00000254719 | 0.456 | 0 | 0 | 0.074 | 0.475 |
| AURKA | TP53 | 9606.ENSP00000216911 | 9606.ENSP00000269305 | 0.096 | 0.875 | 0.9 | 0.935 | 0.999 |
| DDX5 | TP53 | 9606.ENSP00000225792 | 9606.ENSP00000269305 | 0 | 0.748 | 0.9 | 0.988 | 0.999 |
| RPA1 | TP53 | 9606.ENSP00000254719 | 9606.ENSP00000269305 | 0.06 | 0.973 | 0.9 | 0.986 | 0.999 |
| RYR3 | TTN | 9606.ENSP00000373884 | 9606.ENSP00000467141 | 0.149 | 0 | 0 | 0.324 | 0.4 |
| SIRT1 | TP53 | 9606.ENSP00000212015 | 9606.ENSP00000269305 | 0.062 | 0.884 | 0.9 | 0.989 | 0.999 |
| TJP1 | TP53 | 9606.ENSP00000281537 | 9606.ENSP00000269305 | 0 | 0 | 0 | 0.424 | 0.424 |
| TP53 | UBE3A | 9606.ENSP00000269305 | 9606.ENSP00000232165 | 0 | 0.919 | 0.8 | 0.989 | 0.999 |
| TP53 | TTN | 9606.ENSP00000269305 | 9606.ENSP00000467141 | 0 | 0 | 0 | 0.541 | 0.541 |
